# Late presentation of cancer in compound heterozygote PMS2 mutation carrier

**DOI:** 10.1186/1897-4287-9-S1-P38

**Published:** 2011-03-10

**Authors:** Paulien P van Galen, Renee Perrier, Francois P Bernier

**Affiliations:** 1 Division of Clinical Genetics, Alberta Children’s Hospital, Calgary, AB, T3B 6A8, Canada

## Background

Turcot syndrome is clinically characterized by the occurrence of primary brain tumors, colorectal cancer and/or accompanying adenomas. It has been described as both an autosomal dominant and recessive condition and mutations in APC, MLH1, MSH2, MSH6 and PMS2 have been reported. Constitutional Mismatch repair (CMMR) deficiency is a variant of Lynch syndrome (LS) associated with biallelic MMR mutations. Individuals present with NF1 manifestations and generally develop hematological malignancies, brain tumors and/or LS associated cancers, in the first or second decade of life. We report an individual with café au lait macules (CAL) and a history of glioblastoma at 31 and proximal colon cancer at 32. Family history includes a mother with hematologic cancers in her 60’s, maternal half uncle with colon cancer at 48, maternal half uncle with renal cancer and three maternal great uncles with colon cancer.

## Methods

The proband underwent a standard clinical assessment in the cancer genetics clinic.

Immunohistochemistry (IHC) and genetic testing for MLH1, MSH2, MSH6 and PMS2 was completed followed by microsatellite (MSI) studies and imunohistochemistry (IHC) for PMS2.

## Results

IHC on tissue from the patient’s colorectal tumor showed very weak staining of MLH1 in both the tumor and benign colonic mucosa and lymphocytes. No mutation was detected by sequencing and MLPA for MLH1, MSH2 and MSH6. MSI was high and subsequent IHC for PMS2 showed absent staining. Sequencing of PMS2 identified two changes: 2019 delT resulting in a frameshift mutation at codon 673 and 2249G>A (G750D), a missense change in a fully conserved region. In-silico analysis by SIFT [http://sift.jcvi.org/] and Polyphen [http://genetics.bwh.harvard.edu/pph/] predict the missense change to be damaging. This change has also been previously reported as a biallelic mutation in individual with a complete PMS2 gene deletion and history of rectal cancer and brain tumor at 22 and 23 respectively [1]. The proband’s mother is currently being tested to confirm the two PMS2 mutations are in trans.

## Conclusions

We report an individual with Turcot syndrome and biallelic PMS2 mutations who developed her first cancer in her 30’s. In this case, biallelic mutations were suspected due to the history of CALs. This result is in keeping with recent reports suggesting a milder phenotype may exist in individuals with biallelic PMS2 mutations, particularly in those where one mutation may be hypomorphic resulting in some residual MMR proficiency.
